# Distinct Patterns of Myeloid Cell Infiltration in Patients With hrHPV-Positive and hrHPV-Negative Penile Squamous Cell Carcinoma: The Importance of Assessing Myeloid Cell Densities Within the Spatial Context of the Tumor

**DOI:** 10.3389/fimmu.2021.682030

**Published:** 2021-06-14

**Authors:** Tynisha S. Rafael, Hielke M. de Vries, Sarah R. Ottenhof, Ingrid Hofland, Annegien Broeks, Jeroen de Jong, Elise Bekers, Simon Horenblas, Renée X. de Menezes, Ekaterina S. Jordanova, Oscar R. Brouwer

**Affiliations:** ^1^ Department of Urology, Netherlands Cancer Institute, Amsterdam, Netherlands; ^2^ Core Facility Molecular Pathology & Biobanking, Netherlands Cancer Institute, Amsterdam, Netherlands; ^3^ Department of Pathology, Reinier Haga Medisch Diagnostisch Centrum (MDC), The Hague, Netherlands; ^4^ Department of Pathology, Netherlands Cancer Institute, Amsterdam, Netherlands; ^5^ Biostatistics Center, Netherlands Cancer Institute, Amsterdam, Netherlands; ^6^ Center for Gynecologic Oncology Amsterdam (CGOA), Amsterdam University Medical Center, Vrije Universiteit Amsterdam, Amsterdam, Netherlands

**Keywords:** penile cancer, human papillomavirus, squamous cell carcinoma, tumor infiltrating myeloid cells, image analysis, compartments, tumor microenvironment

## Abstract

Comprehensive analysis of tumor infiltrating myeloid cells in the tumor microenvironment of penile squamous cell carcinoma (PSCC) is lacking. In this retrospective study, for the first time, PSCC resection specimens (N = 103) were annotated into the following compartments: intratumoral tumor (IT Tumor), intratumoral stroma (IT Stroma), peritumoral tumor (PT Tumor) and peritumoral stroma (PT Stroma) compartments. We then quantified CD14+, CD68+ and CD163+ myeloid cells within these compartments using an image analysis software and assessed their association with various clinical parameters, including high-risk human papillomavirus (hrHPV) status. In the total cohort, hrHPV status, grade of differentiation, age and tumor size were associated with myeloid cell densities. hrHPV^+^ tumors had higher infiltration rates of CD14+, CD68+ and CD163+ myeloid cells in the IT tumor compartment (p < 0.001, for all) compared to hrHPV^−^ tumors. Furthermore, when examining the association between compartment-specific infiltration and differentiation grade, increased myeloid cell densities in the IT tumor compartment were associated with a more advanced histological grade (p < 0.001, for all). This association remained significant when the hrHPV^−^ cohort (N = 60) was analyzed (CD14+ p = 0.001; CD68+ p < 0.001; CD163+ p = 0.004). Subgroup analysis in the hrHPV^+^ group (N = 43) showed that high infiltration rates of CD68+ and CD163+ cells in the PT tumor compartment were associated with lymph node (LN) metastasis (p = 0.031 and p = 0.026, respectively). Regarding the association between myeloid cell densities and disease-specific survival, the risk of death was found to decrease slightly as the number of myeloid cells in the IT tumor compartment increased (CD14+ p *=* 0.04; CD68+ p = 0.05; CD163+ p = 0.02). However, after adjusting for hrHPV, no independent association between myeloid densities and disease-specific survival were found. Altogether, these findings demonstrate the importance of assessing myeloid cell densities within the spatial context of the tumor. Further studies are needed to unravel the specific phenotype of myeloid cells residing in the different compartments, their effect on clinical parameters and the impact of hrHPV on the recruitment of myeloid cell populations in PSCC.

## Introduction

Penile squamous cell carcinoma (PSCC) is a rare malignancy in developed countries (1, 2). However, the incidence of this mutilating disease is steadily rising in Asian, African and Latin American countries, accounting for up to 10% of malignancies in men (2–4). The prognosis for patients with early stage PSCC is good but worsens with the presence and extent of lymph node metastases (5). Patients with advanced disease have limited treatment options and current cisplatin-based chemotherapy regimens offer poor and non-durable response rates with high toxicity (6, 7). Therefore, there is an unmet need for novel systemic therapeutic strategies that are both effective and tolerable (7).

One of the recognized risk factors of PSCC is a persistent infection with high-risk human papillomavirus (hrHPV), which accounts for 25-40% of all cases (8–11). Importantly, hrHPV–positive (hrHPV^+^) patients tend to have better disease-specific survival (DSS) than hrHPV–negative (hrHPV^−^) PSCC patients (8, 9, 11, 12). The exact molecular background of the observed survival differences is unclear. Recent studies have showed that hrHPV^+^ and hrHPV^−^ tumors are molecularly distinct tumors with different frequency of somatic mutations in p53 pathway genes (13–15). hrHPV^+^ tumors also constitutively express viral oncoproteins E6 and E7 that deregulate the cell cycle (16). As both genomic alterations and HPV-oncogene expression are known to sculpt the tumor microenvironment, the hrHPV-induced survival advantage might be explained by genetic-induced differences in immune reactivity (17, 18).

Tumors can be highly infiltrated with myeloid cells such as plasmacytoid dendritic cells (pDCs), conventional dendritic cells (cDCs), granulocytes, monocytes and macrophages, all of which have multifaceted functions and influence on disease course and ultimately, patient survival (19, 20). Monocytes are usually classified based on the expression of CD14 and CD16 into classical CD14^hi^CD16^−^ and non-classical CD14^+^CD16^hi^ monocytes (21, 22). Macrophages are categorized into the pro-inflammatory M1 phenotype (CD68+ CD80+) and an immunosuppressive M2 (CD68+CD163+) phenotype based on an *in vitro* polarization system (21, 23, 24). However, it should be noted that monocyte/macrophages exhibit extraordinary plasticity *in vivo* and have complex phenotype in the tumor microenvironment beyond this simple categorization (21).

In PSCC, comprehensive analysis of myeloid cell infiltrate is lacking; only limited and conflicting data is available on myeloid cell infiltration (25–27). Several recent studies have highlighted the importance of quantifying immune cell populations within the tissue’s spatial context to properly study the impact of immune infiltrates on tumor progression and, ultimately, clinical outcome (28–31). It has been shown that immune cell populations were not randomly distributed within the tumor microenvironment but were either located in the tumor core and/or near the tumor/stroma border (32–34). Moreover, prognostic and predictive value of immune cells may differ dependent on their location within the tumor. In oropharyngeal carcinomas, for example, compartment-specific myeloid densities were shown to be associated with differing clinical outcome (17, 35, 36).

In this hypothesis-generating study, for the first time, penile squamous cell carcinomas resection specimens tissue structure was annotated into several spatial compartments: intratumoral tumor-, intratumoral stroma-, peritumoral tumor- and peritumoral stroma. We then quantified CD14+, CD68+ and CD163+ myeloid cells within the tissue’s spatial context using an image analysis software and assessed their association with various clinical parameters, including HPV status and patient survival.

## Materials and Methods

### Study Population and Clinical Data Collection

This retrospective study is based on a previously described cohort of patients who were surgically treated for penile carcinoma between 2001 and 2009 at the Netherlands Cancer Institute, Amsterdam, the Netherlands (8, 27, 37). The original cohort consisted of 213 patients of which 52 were hrHPV^+^ and 158 were hrHPV^−^. The hrHPV^−^ patients were chosen to profile the original hrHPV^−^ cohort based on primary tumor pathological T-stage and cause of death. Forty-three hrHPV^+^ cases and 60 hrHPV^−^ cases were selected for further image analysis based on the following inclusion criteria: (1) histologically confirmed diagnosis of invasive penile carcinoma, (2) sufficient and adequate material for tissue image analysis. Clinicopathological characteristics of patients (age, HPV status, primary tumor size, grade of differentiation, staging and presence of lymphovascular invasion (LVI)) were kept in our institutional penile cancer database. Follow-up data providing information on node positivity, disease status and disease-specific mortality were updated from patient medical records. The institutional translational review board approved the acquisition of tissue material.

### hrHPV-Typing

hrHPV status was determined on tissue samples using GP5+/6+ PCR enzyme immunoassay for 14 different HPV subtypes (8). The cocktail contained the following hrHPV subtypes: 16, 18, 31, 33, 35, 39, 45, 51, 52, 56, 58, 59, 66 and 68.

### Immunohistochemistry

Immunohistochemical staining was performed with an automated Ventan**a **Benchmark Ultra autostainer (Ventana Medical Systems, Tucson, AZ, USA). FFPE tissue sections (3 μm) were deparaffinized using the EZ prep solution (Ventana Medical Systems) at 75°C. Next, heat-induced antigen retrieval was carried out using Cell Conditioning 1 (CC1, Ventana Medical Systems) for 64 minutes at 95°C. Slides were then incubated at room temperature for 32 minutes with CD14 (ready-to-use, IgG, EPR3653, Ventana Medical Systems), CD68 (1/20000, IgG1, KP1, DAKO), and CD163 (ready-to-use, IgG1, MRQ-26, Cell Marque). Bound antibody was detected using the UltraView DAB Detection kit (CD163, CD14) or the OptiView DAB Detection Kit (CD68) (Ventana Medical Systems). Slides were finally counterstained with Hematoxylin II and Bluing Reagent (Ventana Medical Systems).

### Image Acquisition and Analysis

Whole-section scans were made using an Aperio Scanner (Leica Biosystems, Solms, Germany). Quantitative analysis of myeloid infiltrates in penile carcinoma were performed (using TissueStudio^®^; Definiens AG, Munich, Germany) and comprised of several steps. First, the original image files (*svs) were loaded and representative intratumoral (IT) and peritumoral (PT) regions of interest (ROIs) were manually selected for each patient. In the next step, trained observers annotated stroma, tumor epithelium and tissue structures not deemed for image analysis, such as necrotic areas and/or areas containing tissue- and/or staining- artifacts. The composer’s machine-learning supervised algorithm then classified the tissue accordingly based on inclusion and exclusion annotations. Next, automatic cell segmentation was performed using a hematoxylin and an IHC (3,3′-Diaminobenzidine) marker threshold to accurately locate and segment cells. The final step was to set the threshold for mean brown chromogen which allowed for the distinguishment of positive from negative cells. The exported results contained the final density for each immune marker (number of positive cells per mm^2^) in the following spatial compartments: peritumoral stroma (PT Stroma), peritumoral tumor epithelium (PT Tumor), intratumoral stroma (IT Stroma) and intratumoral tumor epithelium (IT Tumor). From these data, the final cell densities for the following compartments were calculated: peritumoral (PT Stroma and PT Tumor), intratumoral (IT Stroma and IT Tumor), stroma (PT Stroma and IT Stroma), Tumor (PT Tumor and IT Tumor) and Total (PT Stroma, PT Tumor, IT Stroma and IT Tumor).

### Statistics

Differences in distribution patterns among compartments were studied using Wilcoxon signed-rank test. Correlation between different immune parameters were assessed using spearman correlation analysis using the *ggcorplot* R package. Myeloid cell densities were studied in relation to different clinical parameters, including HPV, lymph node metastasis, T-stage, primary tumor size, grade of differentiation and LVI. The normality of the distributions of continuous variables were examined using the Shapiro–Wilk test and qqplot. Comparisons between groups were performed using the Chi-square test for the categorical variables and Mann–Whitney U test or the Kruskal-Wallis test for continuous variables. Hierarchical clustering analysis was applied based on row-normalized z-scores of myeloid marker densities with the help of the *complexheatmap* R package, using spearman correlation as distance metric and complete-linkage as agglomeration method (38). To study the time to event (DSS) and event type probability, a Fine & Gray survival model taking into account competing risks was used, and for this the R package *cmprsk* was employed (39). All statistical analyses were performed using R (version 4.0.2, Vienna, Austria) and SPSS (version 24, SPSS Inc., Chicago, IL). In all analyses, tests were considered significant for *p*-values < 0.05. Other details, if any, for each experiment are provided within the relevant figure legends.

## Results

### Patient and Clinical Characteristics

The clinicopathological characteristics of the 103 patients stratified by hrHPV status are summarized in **Table 1**. Of these, 43 (33%) were positive for hrHPV^+^ and 60 (58%) were hrHPV^−^. When comparing the hrHPV subgroups with respect to clinical characteristics, we observed a significant difference in grade of differentiation (p = 0.014). Interestingly, even though hrHPV^−^ tumors were mostly grade 1 tumors (43% *vs* 16%), hrHPV^−^ patients had worse DSS than hrHPV^+^ counterparts, with 11 (18%) and 1 (2%) PSCC related deaths, respectively (p = 0.038). Among hrHPV^+^ tumors, HPV16 was the predominant type, followed by HPV18.

**Table 1:** Patient characteristics and clinical parameters of the cohort stratified according to hrHPV status.

	Total *N* = 103 (%)	hrHPV^−^ * N* = 60 (%)	hrHPV^+^ * N* = 43 (%)	*p*-Value*
Age median (IQR)	66.9 (57.2-74.4)	69.3 (58.8-74.6)	63.6 (54.4-71.2)	0.074
Tumor size median (IQR)	3.0 (1.9-4.0)	3.0 (2.0-4.5)	2.5 (1.5-4.0)	0.240
**pT-stage**				0.810
pT1a-b	30 (29.1)	16 (26.7)	14 (32.6)	
pT2	63 (61.2)	38 (63.3)	25 (58.1)	
pT3-4	10 (9.7)	6 (10.0)	4 (9.3)	
**Grade of differentiation**				**0.014**
Grade 1	33 (32.0)	26 (43.3)	7 (16.3)	
Grade 2	52 (50.5)	26 (43.3)	26 (60.5)	
Grade 3	18 (17.5)	8 (13.3)	10 (23.3)	
LVI	14 (13.6)	8 (13.3)	6 (14.0)	0.928
pN-stage 1 to 3	35 (34.0)	21 (35.0)	14 (32.6)	0.796
Death by penile cancer	12 (11.7)	11 (18)	1 (2)	**0.038**†

IQR, interquartile range; hrHPV, high risk human papilloma virus; LVI, lymphovascular invasion; pN-stage, pathological nodal stage.

*Comparing the two hrHPV subgroups. Chi-square test or Fisher’s exact test for the categorical variables and Mann–Whitney U test for the continuous variables.

^†^Competing risk survival model.

Bold numbers are statistically significant.

### Differential Distribution of Myeloid Cell Populations Across Stroma- and Tumor Compartments

**Figure 1:**
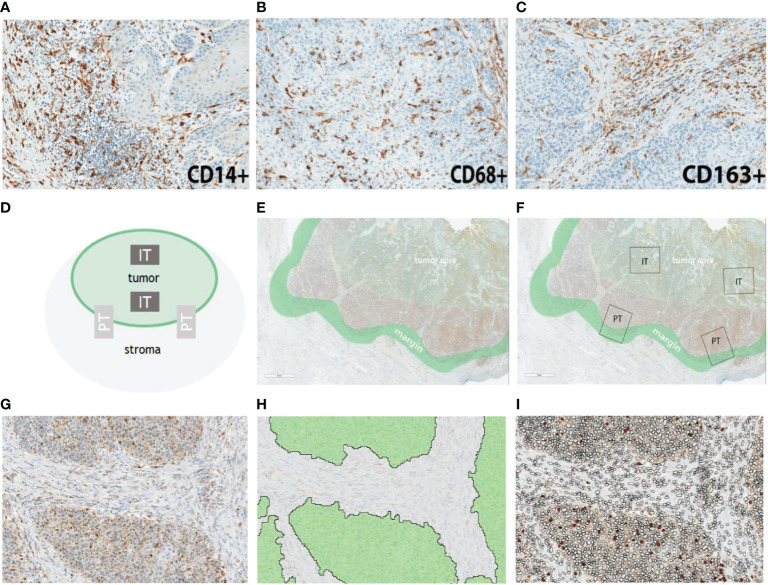
Representative examples of myeloid cell infiltration rates for CD14 **(A)**, CD68 **(B)** and CD163 **(C)** in penile cancer. Semiautomatic analysis pipeline. **(D)** Schematic representation of the regions of interest. **(E)** Representative definition of tumor core and tumor margin. **(F)** In situ representation of two intratumoral (IT) and two peritumoral (PT) regions which were selected for marker quantification. **(G)** Example of an IT compartment. **(H)** Segmentation of the IT compartment into IT Tumor (green) and IT Stroma (grey). **(I)** IT Stroma and IT Tumor compartments were infiltrated by myeloid cells (filled circles). Scale bar is 2 mm.

Representative examples of the immunohistochemical staining results are shown in **Figure 1**. In order to detect potential differences in infiltration rates in PSCC, CD14+ (**Figure 1A**), CD68+ (**Figure 1B**) and CD163+ (**Figure 1C**) cell populations were quantified and compared between the following spatial compartments: intratumoral tumor (IT Tumor), intratumoral stroma (IT Stroma), peritumoral tumor (PT Tumor) and peritumoral stroma (PT Stroma) (**Figures 1D-I**).

**Figure 2:**
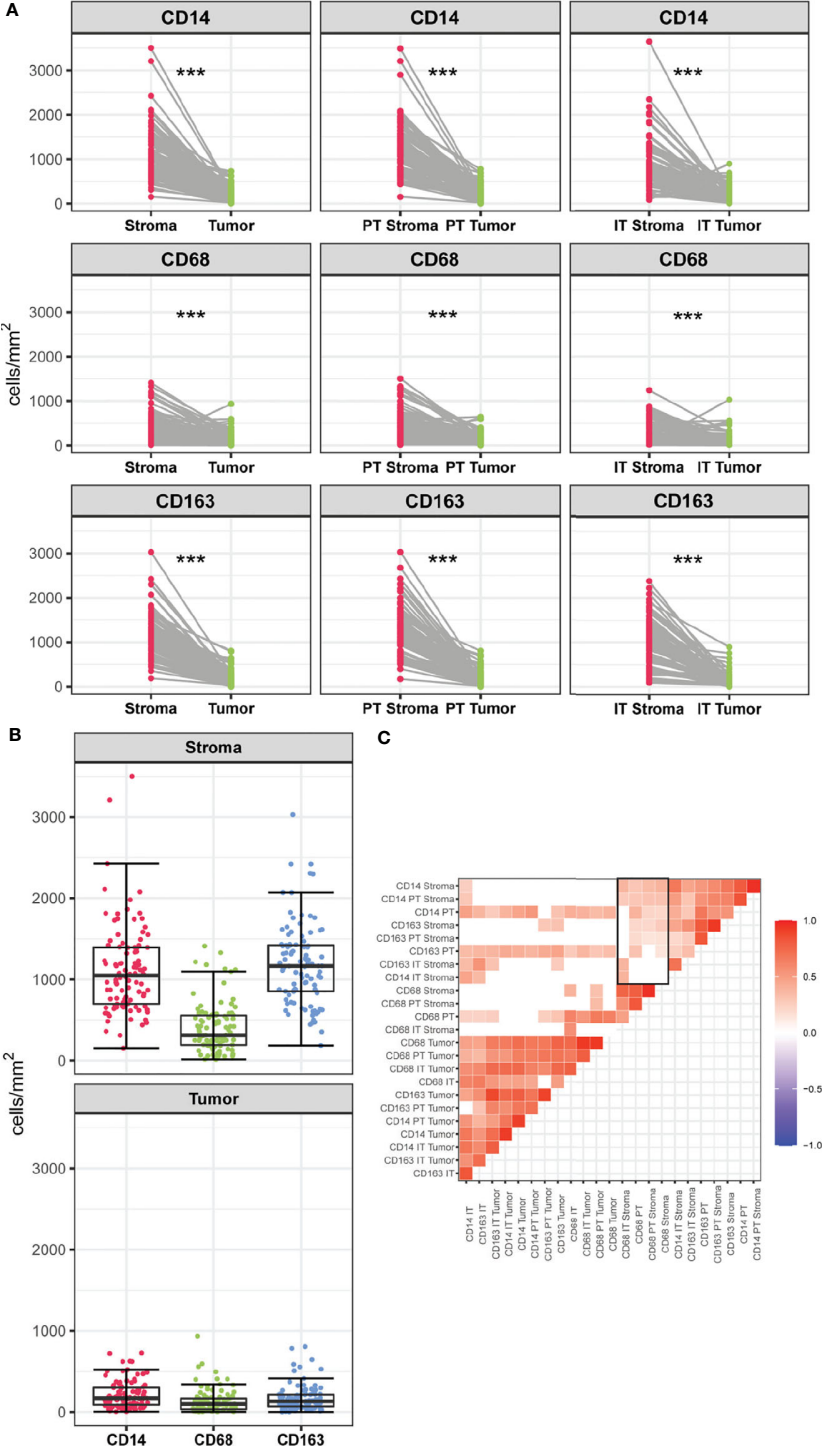
**(A)** Infiltration rates of CD14+, CD68+ and CD163+ cells (cells/mm^2^) in stromal versus tumoral compartments in PSCC. Myeloid densities in the stromal and tumoral compartments were compared using Wilcoxon-Signed Rank Test, ***p < 0.001. **(B)** Infiltration rates of CD14+, CD68+, CD163+ (cells/mm^2^) within the stromal and tumor compartment. **(C)** Correlation matrix of myeloid cell populations measured in spatial compartments. Blank spaces indicate no significant coefficients and colors correspond to the direction of the correlation (red for positive, blue for negative). Rectangle indicates weak to moderate correlations between densities of CD68+ cells and CD14+ and CD163+ cells in the stromal compartments.

Myeloid cell densities differed between spatial compartments, with stromal compartments containing higher numbers of CD14+, CD68+ and CD163+ cells than the tumoral compartments (**Figure 2A**). Further compartmentalization showed similar results; myeloid cell populations displayed the highest density in the PT Stroma and IT Stroma compartments. Comparing the myeloid cell populations, lower number of CD68+ cells were found compared to CD14+ and CD163+ in the stroma compartment (median of cells/mm2 311.4, 1047.1, and 1163.6, respectively), whereas densities in the tumor compartment were comparable (median of cells/mm2 97.3, 171.4, and 132.4, respectively) (**Figure 2B**). This is also reflected in the correlation matrix of myeloid cell densities measured in all spatial compartments (**Figure 2C**). Moderate to strong correlations were observed between CD14+, CD68+ and CD163+ cell densities in the tumoral compartments. The distribution of CD14+ infiltrating cells followed that of CD163+ in all the stromal compartments. Interestingly, weaker correlations were observed between densities of CD68+ cells and CD14+ and CD163+ cells in the stromal compartments (**Figure 2C**) highlighting the contribution of spatial distribution.

### Density and Spatial Distribution of CD14+, CD68+, CD163+ Myeloid Cells in PSCC: Association With Clinicopathological Parameters

**Figure 3:**
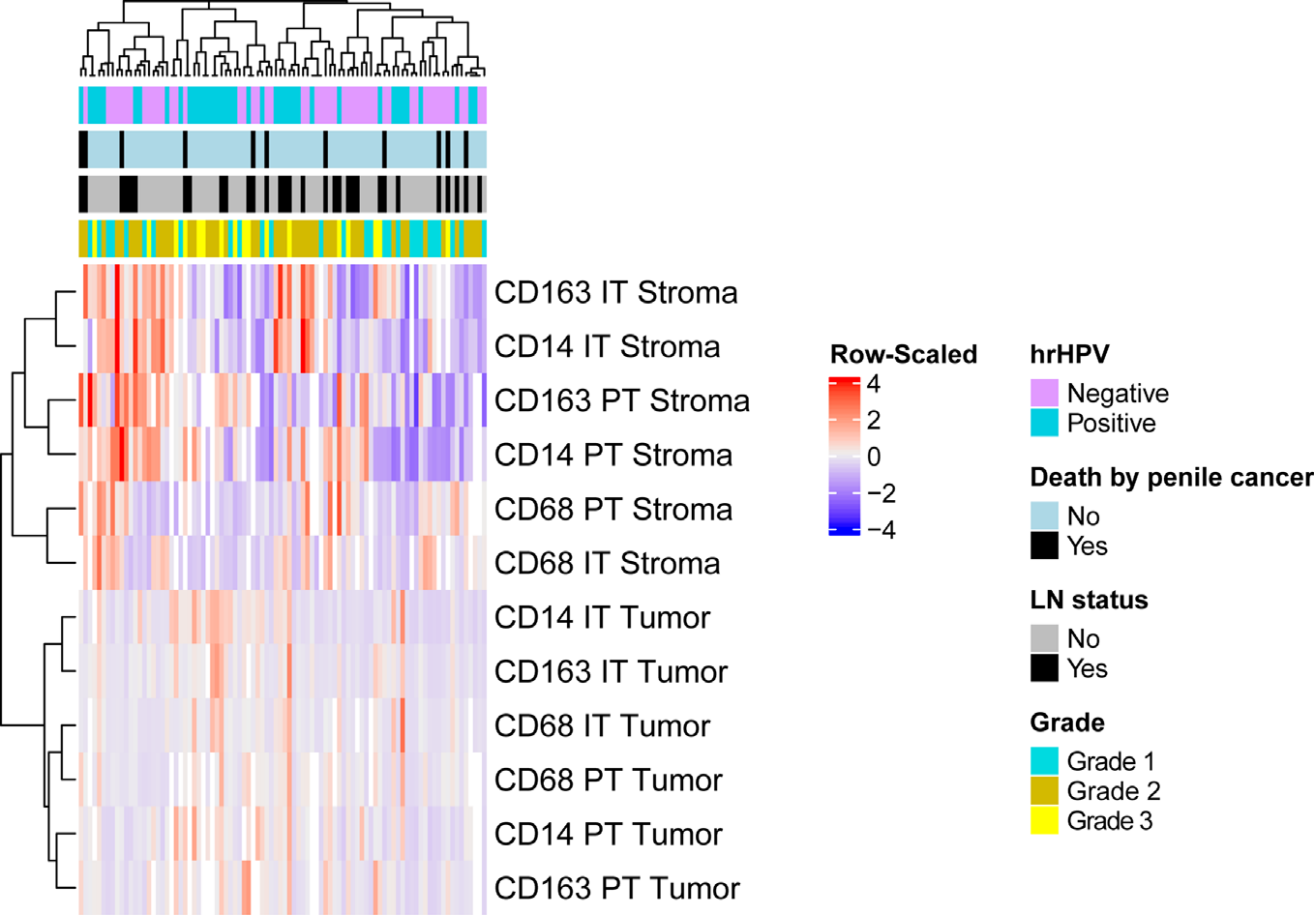
Unsupervised clustering analysis using spearman correlation distance and complete linkage were conducted based on the infiltration rates of myeloid cells. The vertical axis represents patient samples, and the horizontal axis represents myeloid cell infiltration in different compartments. Each colored square in the heatmap represents the Z-score for the presence of one marker in one compartment, with the highest cell number rates shown in red, mean in grey and lowest in blue. Missing values are shown in white. Top horizontal bars are clinical annotations of the samples indicating the following histopathological characteristics: hrHPV status, survival status, LN status and grade. hrHPV status, high-risk human papilloma virus status; LN status, lymph node status; IT Tumor, intratumoral tumor; IT Stroma, intratumoral stroma; PT Tumor, peritumoral tumor; PT Stroma, peritumoral stroma.

We performed unsupervised hierarchical clustering of 90 samples to explore myeloid marker profiles and their association with clinicopathological features (**Figure 3**). Thirteen patients were excluded from clustering analysis due to missing values. Some patients displayed increased CD14+, CD68+ and CD163+ infiltration rates in the stromal compartments but not in the tumoral compartments, while the opposite was observed for others. These differences in infiltration rates between samples, however, could not be explained by clinicopathological parameters, as samples did not cluster according to clinical features. Further in-depth myeloid infiltration analysis was then performed to determine the associations between compartment-specific CD14+, CD68+ and CD163+ cell densities and clinical parameters.

**Figure 4:**
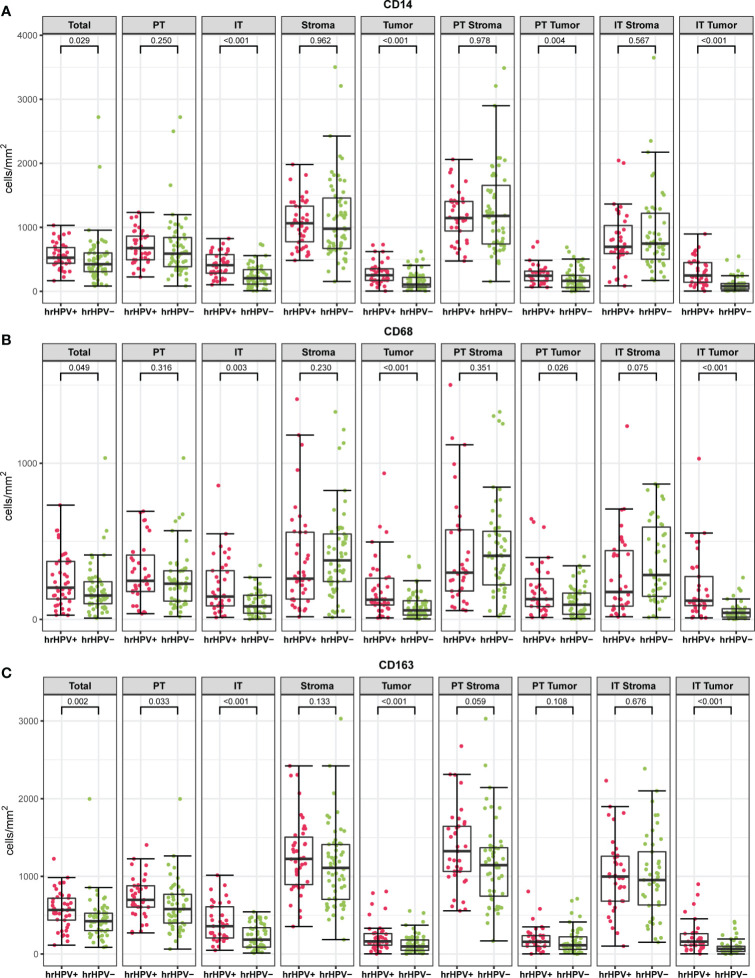
The boxplots indicate myeloid cell densities (cells/mm^2^) of CD14 **(A)**, CD68 **(B)** and CD163 **(C)** in different spatial compartments stratified according to hrHPV status (Mann-Whitney U test). PT, peritumoral; IT, intratumoral; hrHPV^+^, high risk human papilloma virus positive specimen; hrHPV^−^, high-risk human papilloma virus negative specimen.

Interestingly, the hrHPV^+^ tumors had a markedly different myeloid cell infiltration profile compared to the hrHPV^−^ tumors. The density of CD14+, CD68+, CD163+ cells in the intratumoral tumor (IT Tumor) compartment was significantly higher in hrHPV^+^ tumors than in hrHPV^−^ tumors (p < 0.001, p < 0.001, p < 0.001, respectively) (**Figure 4** and **Supplementary Table 1**). This correlation, however, was not apparent in the intratumoral stroma (IT Stroma) compartment (p = 0.57, p = 0.08, p = 0.68, respectively). Further compartmentalization of the peritumoral (PT) compartment into PT Tumor and PT Stroma, showed that CD14+ and CD68+ cell densities differed significantly between hrHPV subgroups only when the PT Tumor compartment was taken into account, with increased numbers in the hrHPV^+^ subgroup (p = 0.004, p = 0.026, respectively) (**Figures 4A, B**). Overall, differences in myeloid cell densities between hrHPV^+^ and hrHPV^−^ tumors were found to be mainly driven by the IT Tumor compartment and to some degree by the PT Tumor compartment.

**Figure 5:**
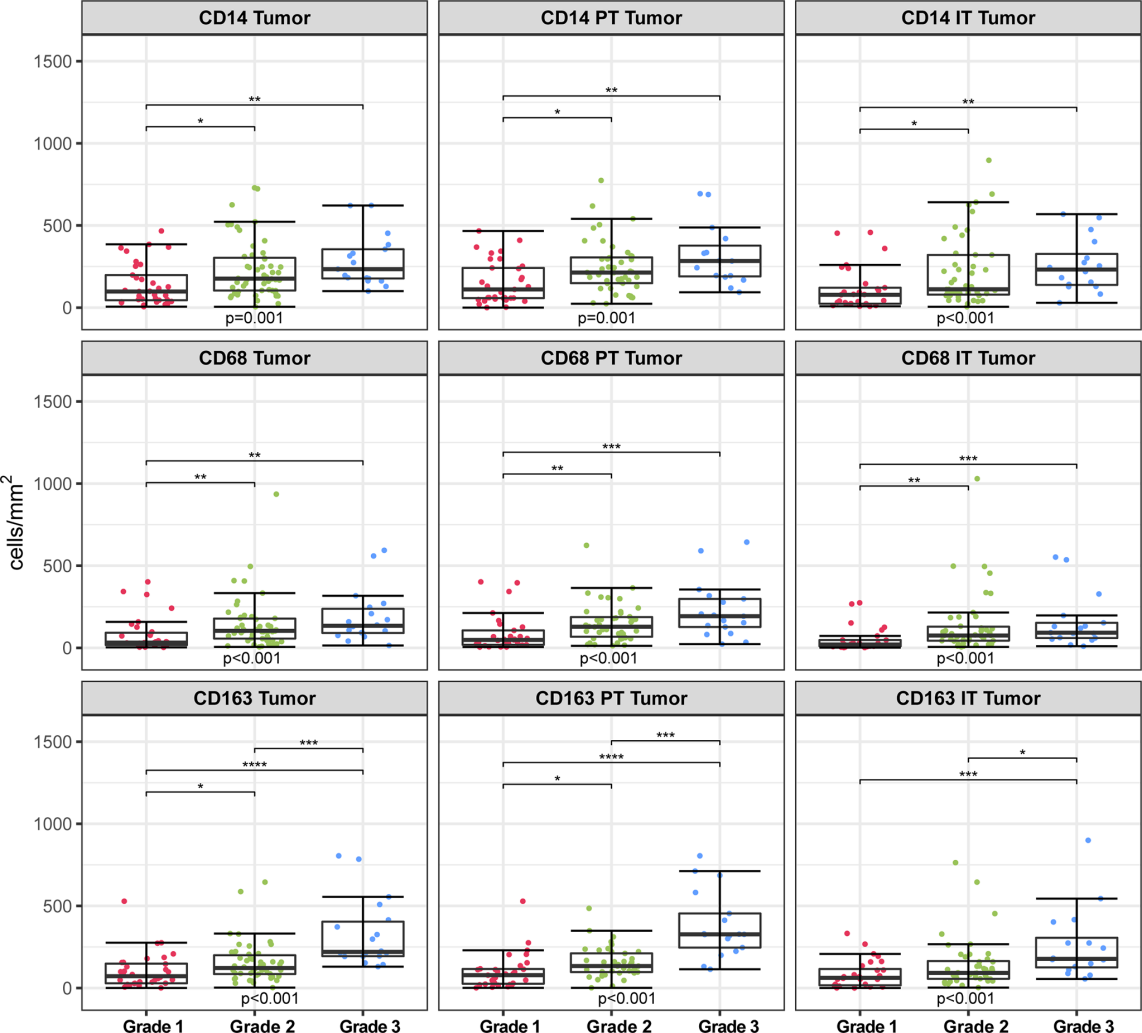
Infiltration rates of CD14+, CD68+, CD163+ cells (cells/mm^2^) in different spatial compartments stratified according to differentiation grade. Kruskal-Wallis test with multiple comparisons between differentiation grade groups (Dunn’s tests with Bonferroni correction). *p < 0.05, **p < 0.01, ***p < 0.001, ****p < 0.0001. PT, peritumoral; IT, intratumoral.

Significant differences were found between hrHPV^−^ and hrHPV^+^ subgroups in terms of grade of differentiation and myeloid infiltration, with the hrHPV^−^ counterparts having more grade 1 tumors and reduced myeloid cell infiltration in the tumoral compartments. In addition, myeloid cell densities in the tumor compartment were found to differ significantly based on differentiation grade (CD14 p = 0.001; CD68 p < 0.001; CD163 p < 0.001) (**Figure 5** and **Supplementary Table 1**). Further compartmentalization of the tumor compartment into PT Tumor (CD14 p = 0.001; CD68 p < 0.001; CD163 p < 0.001) and IT Tumor (CD14 p < 0.001; CD68 p < 0.001; CD163 p < 0.001) showed similar results. The prevalence of CD14+, CD68+ and CD163+ cells was higher in grade 3 tumors than in grade 1 tumors, both in PT Tumor and IT Tumor compartments. CD163+ cell numbers also differed significantly between grade 2 and grade 3 tumors, with the latter having increased myeloid cell infiltration in the Tumor, PT Tumor and IT Tumor compartments.

In addition, weak and moderate correlations were found between stromal compartment-specific infiltration rates and clinical parameters, including age and tumor size (**Supplementary Table 1**). No associations were found between myeloid cell densities and lymph node status, T-stage and LVI in the whole cohort (**Supplementary Table 1**).

#### Subgroup Analyses

We also investigated the associations between myeloid marker densities and clinicopathological parameters separately across hrHPV^−^ and hrHPV^+^ subgroups.

In hrHPV^−^ (N = 60), myeloid densities in the tumoral compartments were associated with grade of differentiation. CD14+, CD68+ and CD163+ cell densities in the intratumoral tumor (IT tumor) compartment (p = 0.001; p < 0.001 and p = 0.004, respectively) and peritumoral tumor (PT Tumor) compartment (p = 0.001; p = 0.003 and p < 0.001, respectively) differed between grades of differentiation. Interestingly, in the hrHPV^+^ subgroup (N = 43) high infiltration rates of CD68+ and CD163+ cells in the PT Tumor compartment were associated with LN metastasis (p = 0.031 and p = 0.026, respectively). High infiltration rates of CD163+ cells in the PT tumor compartment were also associated with grades of differentiation (p = 0.021) and tumor stage (p = 0.030).

### Prognostic Significance of CD14+, CD68+ and CD163+ Infiltrating Cells in PSCC

Patients included in this cohort had an average age of 67 years and a large majority (37%) died due to causes not related to penile cancer. Therefore, we performed a survival data analysis to estimate the cumulative incidence function of the event (DSS), allowing for competing risks; that is, we consider as main event death due to penile cancer while correcting for death due to other causes. The survival models were fitted with and without hrHPV status, which is one of the most important clinical prognostic factors for PSCC. For a reference, we also fitted the model of survival with hrHPV status; hrHPV status had a significant positive effect on survival (sHR: 0.12, 95% CI [0.02–0.89], p = 0.038).

We next determined the association between myeloid marker compartment-specific densities (analyzed as continuous variables) and survival in the whole cohort consisting of both hrHPV^−^ and hrHPV^+^ patients. (**Table 2**). Although myeloid cell densities in the total compartment were not associated with survival (CD14 p *=* 0.51; CD68 p = 0.30; CD163 p = 0.13), when analyzed according to localization, the prognostic value of CD14+, CD68+ and CD163+ could be appreciated. The risk of death was found to decrease slightly but in a statistically significant manner as the number of CD14+ (sHR: 0.997, 95% CI [0.993-1], p = 0.04), CD68+ (sHR: 0.995, 95% CI [0.991-1], p = 0.05) and CD163+(sHR: 0.995, 95% CI [0.999-1], p = 0.02) myeloid cells in the intratumoral tumor (IT Tumor) compartment. However, after adjusting for hrHPV status, myeloid marker densities in the IT Tumor compartment were no longer significantly associated with survival (CD14 p = 0.65, CD68 p = 0.96, CD163 p = 0.53).

**Table 2:** The Fine and Gray proportional subdistribution hazard models for penile cancer-specific death in the whole cohort.

Single-covariate model	CD14		CD68		CD163	
	sHR* [CI]	*p*-Value	sHR* [CI]	*p*-Value	sHR* [CI]	*p*-Value
Total	0.999 [0.997-1]	0.51	0.999 [0.996-1]	0.3	0.998 [0.996-1]	0.13
Tumor	0.999 [0.996-1]	0.5	0.998 [0.995-1]	0.18	0.999 [0.997-1]	0.35
Stroma	1 [0.999-1]	0.84	1 [0.999-1]	0.53	0.999 [0.998-1]	0.4
IT	0.997 [0.994-1]	**0.027**	0.997 [0.993-1]	0.11	0.997 [0.993-1]	0.068
PT	1 [0.998-1]	0.77	1 [0.998-1]	0.91	0.999 [0.997-1]	0.35
IT Tumor	0.997 [0.993-1]	**0.04**	0.995 [0.991-1]	**0.05**	0.995 [0.999-1]	**0.022**
IT Stroma	0.999 [0.998-1]	0.38	1 [0.999-1]	0.49	1 [0.998-1]	0.84
PT Tumor	1 [0.997-1]	0.83	1 [0.997-1]	0.99	1 [0.999-1]	0.32
PT Stroma	1 [0.999-1]	0.98	1 [0.999-1]	0.59	0.999 [0.998-1]	0.43
**Multiple covariates model**		***p*-Value**		***p*-Value**		***p*-Value**
IT Tumor	0.999 [0.996-1.00]	0.65	1 [0.994-1.01]	0.96	0.998 [0.993-1.00]	0.53
hrHPV status	0.145 [0.016-135]	0.09	0.12 [0.009-1.62]	0.14	0.16 [0.018-1.42]	0.10

IT, intratumoral; PT, peritumoral; sHR, subdistribution hazard ratio; CI, 95% confidence interval; hrHPV, high-risk human papilloma virus; LN, lymph node.

The subdistribution hazard model of the Fine and Gray method was used for single-covariate and multiple-covariate analysis of penile cancer-specific death. The subdistribution hazard ratios (sHRs) obtained from the models describe the effect of covariates on the incidence of penile-cancer death after accounting for competing events.

^*^Subdistribution hazard ratio for each point increase in myeloid-cell counts.

Bold numbers are statistically significant.

#### Subgroup Analyses

These analyses were repeated in the hrHPV^−^ subgroup (N = 60) and no associations were found between myeloid marker densities, compartmentalization and survival (**Supplementary Table 2**).

In the hrHPV^+^ subgroup (N = 43), only one hrHPV^+^ patient died due to penile cancer making it impossible to perform meaningful risk analysis in this cohort.

## Discussion

Current immunotherapy trials are aimed at combating tumor-promoting myeloid cells which, in many tumor types, are key contributors to the tumor microenvironment composition. These tumor-promoting myeloid cells sustain an immunosuppressive environment forming roadblocks for current cancer therapies, including checkpoint inhibitors (40). Patient selection based on the type and infiltration rate of tumor residing myeloid cells may therefore lead to improved response to immunotherapy (19).

With the advancing immunotherapeutic options in mind, our group has extensively studied the tumor microenvironment in hrHPV^+^ and hrHPV^−^ tumors PSCC over the past decade (8, 27, 37, 41, 42). We and others have shown that hrHPV^+^ and hrHPV− PSCC tumors are two immunologically distinct subgroups with different levels of immune cell infiltration and immunosuppression (25–27, 37, 43). An increased tumoral PD-L1 immune-checkpoint expression was observed in hrHPV^−^ compared to hrHPV^+^ tumors (49% versus 32%; *P* = 0.03) (27, 37). Furthermore, higher density of stromal cytotoxic T cells and stromal granzyme B were observed in HPV^+^ cases compared to HPV^-^ (25, 26, 43). This observation, however, was not apparent in our previous study (27). Regarding myeloid cells, only a few studies have investigated myeloid cell infiltrates in penile cancer and no differences in myeloid numbers were found between hrHPV^+^ and hrHPV^−^ cases (25–27). However, these studies only quantified myeloid cells within the tumor and stroma compartments without distinguishing the specific location within the tumor (e.g., tumor nest or periphery).

The compartmentalization of the tumor tissue is of importance when studying the impact of immune cell infiltration on disease pathology, progression and treatment response (29, 30). To our knowledge, this is the first study in penile cancer that reports on the distribution patterns of tumor infiltrating myeloid cells and the associations between compartment-specific myeloid cell densities and clinical parameters. Ultimately, a comprehensive analysis and understanding of tumor-promoting myeloid cells within their spatial context may lead to novel immunotherapeutic strategies in PSCC.

In the present hypothesis-generating study, significant differences were observed between hrHPV^+^ and hrHPV^−^ tumors, when assessing myeloid cell populations within their spatial context. hrHPV^+^ tumors had markedly higher numbers of CD14+, CD68+ and CD163+ myeloid cells in the intratumoral tumor (IT Tumor) compartment compared to hrHPV^−^ tumors (p=0.001, p < 0.001, p=0.002, respectively). This observation, however, is not unique for penile cancer. Higher densities of CD68+ and CD163+ cells were also observed in HPV^+^ tumors of other HPV-induced squamous cell carcinomas, such as oropharyngeal squamous cell carcinoma and cervical squamous cell carcinoma (17, 36, 44, 45). These findings could have important implications for the design of effective immunotherapeutic strategies, as immune lineages that are different between disease entities may require specific targeting (46). Since hrHPV^+^ patients had high numbers of tumor-infiltrating myeloid cells and presumably a predominance of the immunosuppressive CD68+ CD163+ M2 phenotype, they may require a combination of myeloid cell modulator and an inhibitor of PD-1/PD-L1 axis to overcome the influence of immunosuppressive cells and ultimately enhance anti-tumor response (47–49). Furthermore, hrHPV^+^ patients with aggressive tumors, may benefit from such combinatorial therapy, as we showed a probable association between high infiltration rates of CD68+ and CD163+ cells in the peritumoral tumor (PT Tumor) and lymph node metastasis. Because lymph node metastasis is the major cause for penile cancer-related deaths, we considered that CD163+ macrophages may have a clinical significance as a prognostic factor in patients with hrHPV^+^ PSCC. However, here we failed to show a link between CD163+ myeloid infiltration rates and survival in the hrHPV^+^ subgroup, probably due to the low sample size (N=43) and event rate.

Presently, it is not clear what causes such distinct patterns of myeloid cell infiltration between hrHPV^+^ and hrHPV^−^ tumors. Emerging evidence suggests that genomic alterations supporting the development of cancer can dictate the immune contexture of tumors (50). In line with the above, we hypothesized that significant differences in myeloid cell numbers may be related to the two distinct etiologies of PSCC: the hrHPV-dependent and hrHPV-independent carcinogenesis. Moreover, growing efforts to identify genomic alterations implicated in the two pathways of PSCC development have expanded our knowledge on mutational patterns. Recent studies have shown that the frequency of mutations was mostly similar between HPV^+^ and HPV^−^ PSCC, with some exceptions, such as mutations in p53 pathway genes (P53, CHECK2, *CDKN2A*), which were commonly mutated in HPV^−^ but not in HPV^+^ PSCC (13, 14). Similar to PSCC, HPV^+^ vulvar and head and neck cancers exhibit lower frequency of TP53 mutations than HPV^−^ tumors (13, 14, 51–56). This owes mainly to the action of E6 in eliminating p53 leaving HPV^+^ cancer cells with less evolutionary pressure to select for p53 mutations (57). Moreover, in several tumor models, loss or loss-of-function mutation of p53 activated the NF-κB pathway, changing the secretome of cancer cells and thereby modulating the tumor microenvironment by recruiting monocytes and macrophages (50). In hrHPV^+^ PSCC, loss of p53 may also prolong NF-κB activation and consequently change the secretome of HPV+ cancer cells, inducing the chemokine-dependent recruitment of macrophages and/or polarization of macrophages toward a tumor-promoting CD68+CD163+ M2 phenotype. Further research in a larger cohort is needed to study the mutational landscape of hrHPV^+^ and hrHPV^−^ PSCC and to determine how mutational signatures can influence the secretome and consequently the myeloid component of these tumors. Furthermore, from a clinical perspective, better insights into what drives myeloid cell infiltration can help identify novel targets for anti-cancer immunomodulatory therapies.

Myeloid cell infiltration patterns were also associated with grade of differentiation, with higher myeloid cell infiltration rates found in tumors with a more advanced histological grade. This has been previously described in oropharyngeal, bladder and triple negative breast cancers, but the underlying mechanism is still unknown (58–61). The association between myeloid infiltration patterns and grade of differentiation remained significant when the hrHPV^−^ subgroup was analyzed separately. These findings raise the question whether hrHPV^−^ tumors were weakly infiltrated compared to hrHPV^+^ tumors because they were mostly grade 1 tumors or that they were mostly grade 1 tumors because of the less extensive myeloid infiltration. The latter suggests a potential role of myeloid cells in tumor progression. In cervical and oropharyngeal cancer, for example, high infiltration by CD163+ and CD68+ macrophages was correlated with the tumor progression from normal tissue to dysplasia to carcinoma (45, 49). Additional studies are needed to investigate whether myeloid cells can serve as potential marker for disease progression and elucidate the potential mechanism by which myeloid cell infiltration patterns promote penile cancer progression.

Next, competing risk models were performed to study the association between myeloid cell densities, compartmentalization and survival. When CD14+, CD68+ and CD163+ myeloid densities in the intratumoral tumor (IT Tumor) increased, the risk of death was found to decrease slightly but in a statistically significant manner. However, no significant associations were found between myeloid densities and survival after adjusting for hrHPV status. This could be explained by the fact that hrHPV had a strong association with both survival and myeloid cell densities; hrHPV^+^ patients had a more favorable survival than hrHPV^−^ counterparts and higher levels of myeloid cell densities in the intratumoral tumor (IT Tumor) compartment. These results were not in accordance with other studies in HPV-induced carcinomas, showing that macrophages number was an independent prognostic factor (44, 62). However, both these studies used cut-off points for creating subgroups for survival analysis and cut-off points are generally data-specific and may not be as reproducible and applicable in a clinical setting as using the continuous data.

A major strength of this study is that myeloid cell infiltration rates were quantified within the spatial context of the tumor using robust image analysis algorithms. However, in addition to determining compartment-specific infiltration, it is important to determine the phenotype of immune cell subpopulations residing in the different tumor compartments. Most studies on myeloid infiltrates have classified myeloid cells into monocytes, tumor associated macrophages (TAMs), tumor-associated granulocytes, myeloid-derived suppressor cells (MDSCs) or monocyte-derived dendritic cells (20, 63). The IHC methodology used here, however, did not allow for the identification of different myeloid cell subsets. Interestingly, we found relatively lower CD68+ myeloid densities in the stromal compartments compared to CD163+ infiltrating cells. One explanation could be that a considerably higher number of single positive CD163+ cells was present in the stromal compartment compared to CD68+CD163+ cells. In OPSCC, for example, higher numbers of single positive CD163+ cells were observed in the stroma compared to double-positive CD68+CD163+ cells and single positive CD68+ cells (64). Similarly, in the tumor microenvironment of HPV16+ OPSCC patients, CD14‒CD33‒CD163+ cell populations were found to consist out of CD14‒CD68+CD163+ M2 macrophages and an underappreciated population of CD14‒CD68‒CD163+ CD1c+ conventional dendritic cell type 2 (65). These cells produce high levels of IL-12 and IL-18 cytokines, which are required for type 1 T cell responses, and have been correlated with prolonged survival (65). Further characterization of the specific myeloid cell subpopulations in PSCC is needed and a solution to that problem could be the application of high-dimensional techniques, such as multiplex immunofluorescence and/or imaging mass cytometry, that enable simultaneous spatial analysis of multitude of cell types, which is critical for understanding the tumor-immune system cross-talk (66–70). Therefore, our group is currently aiming to comprehensively characterize the tumor microenvironment of hrHPV^+^ and hrHPV^−^ PSCC tumors using the above-mentioned high-dimensional technologies.

Another limitation of this study is that no correction for multiple testing was applied. We did not perform multiple testing correction to avoid the chance of dramatically increasing the risk of committing type II errors and therefore decreasing the possibility of detecting real differences (71). Since knowledge on the tumor microenvironment in penile cancer is scarce and there is unmet need for new treatment strategies, we believe further confirmatory studies in another cohort are needed to confirm the results of this hypothesis-generating study.

In conclusion, the assessment of myeloid cell densities within their spatial context seems to be essential for identifying immune microenvironment differences between hrHPV^−^ and hrHPV^+^ PSCC, with the latter having higher CD14+, CD68+ and CD163+ myeloid cell infiltration in the intratumoral tumor (IT Tumor) compartment. Further confirmatory studies are needed to not only validate our results but also to characterize the specific myeloid cell subpopulations residing in the tumoral compartments and understand the impact of HPV on their recruitment in hrHPV^+^ PSCC. Ultimately, this will lead to better understanding of the tumor microenvironment of this rare tumor type and may help improve selection of PSCC patients for specific myeloid cell-targeting immunotherapy trials.

## Data Availability Statement

The raw data supporting the conclusions of this article will be made available by the authors, without undue reservation.

## Ethics Statement

The studies involving human participants were reviewed and approved by NKI translational review board. Written informed consent for participation was not required for this study in accordance with the national legislation and the institutional requirements.

## Author Contributions

This study was designed by EJ, SH, and OB. Image analysis was done by TR and EJ, and revised by EB and JJ. Clinicopathological data were collected by SO and HV, and parts of it were revised by JJ. IH and AB performed immunohistochemistry stainings. Statistical analysis was done by TR and RM. The manuscript was drafted by TR, and sections of it were written by HV, EJ, and OB. All authors contributed to the article and approved the submitted version.

## Conflict of Interest

The authors declare that the research was conducted in the absence of any commercial or financial relationships that could be construed as a potential conflict of interest.
