# Supplementary material for: Distinct Patterns of Myeloid Cell Infiltration in Patients With hrHPV-Positive and hrHPV-Negative Penile Squamous Cell Carcinoma: The Importance of Assessing Myeloid Cell Densities Within the Spatial Context of the Tumor
Source: Front Immunol. 2021 Jun 14;12:682030. doi: 10.3389/fimmu.2021.682030 (PMC8236714; doi:10.3389/fimmu.2021.682030)
Supplement: Supplementary file 2 [file Table_2.docx]

Supplementary Material

| **Table 2.** The Fine and Gray proportional subdistribution hazard models for penile cancer-specific death in the hrHPV^-^ cohort. | | | | | | |
| --- | --- | --- | --- | --- | --- | --- |
|  | CD14 | | CD68 | | CD163 | |
| **Single-covariate model** | sHR* [CI] | *p*-Value | sHR* [CI] | *p*-Value | sHR* [CI] | *p*-Value |
| Total | 1 [0.998-1] | 0.67 | 0.999 [0.997-1] | 0.45 | 0.999 [0.997-1] | 0.43 |
| Tumor | 1 [0.998-1] | 0.48 | 1 [0.997-1] | 0.72 | 1 [0.999-1] | 0.66 |
| Stroma | 1 [0.999-1] | 0.62 | 1 [0.998-1] | 0.56 | 0.999 [0.998-1] | 0.16 |
| IT | 0.999 [0.996-1] | 0.59 | 0.999 [0.995-1] | 0.75 | 0.999 [0.995-1] | 0.69 |
| PT | 1 [0.999-1] | 0.79 | 1 [0.998-1] | 0.7 | 0.999 [0.998-1] | 0.45 |
| IT Tumor | 1 [0.996-1] | 0.87 | 1 [0.996-1] | 0.41 | 0.998 [0.994-1] | 0.5 |
| IT Stroma | 0.999 [0.998-1] | 0.27 | 1 [0.998-1] | 0.82 | 1 [0.998-1] | 0.88 |
| PT Tumor | 1 [0.998-1] | 0.39 | 1 [0.997-1] | 0.65 | 1 [0.999-1] | 0.35 |
| PT Stroma | 1 [0.999-1] | 0.85 | 1 [0.998-1] | 0.58 | 0.999 [0.998-1] | 0.25 |
| IT: intratumoral; PT: peritumoral; sHR: subdistribution hazard ratio; CI: 95% confidence interval; hrHPV: high-risk human papilloma virus; LN: lymph node.  The subdistribution hazard model of the Fine and Gray method was used for single-covariate and multiple-covariate analysis of penile cancer-specific death. The subdistribution hazard ratios (sHRs) obtained from the models describe the effect of covariates on the incidence of penile-cancer death after accounting for competing events.  ^*^ Subdistribution hazard ratio for each point increase in myeloid-cell counts. | | | | | | |

.
